# Notch signaling in female cancers: a multifaceted node to overcome drug resistance

**DOI:** 10.20517/cdr.2021.53

**Published:** 2021-08-05

**Authors:** Maria V. Giuli, Angelica Mancusi, Eugenia Giuliani, Isabella Screpanti, Saula Checquolo

**Affiliations:** ^1^Laboratory of Molecular Pathology, Department of Molecular Medicine, Sapienza University, Rome 00161, Italy.; ^2^Scientific Direction, San Gallicano Dermatological Institute IRCCS, Rome 00144, Italy.; ^3^Department of Medico-Surgical Sciences and Biotechnology, Sapienza University, Latina 04100, Italy.; ^4^Center for Life Nano Science@Sapienza, Istituto Italiano di Tecnologia, Rome 00161, Italy.

**Keywords:** Notch signaling, drug resistance, female-specific cancers, cancer stem cells, epithelial-to-mesenchymal transition, drug efflux, tumor microenvironment

## Abstract

Drug resistance is one of the main challenges in cancer therapy, including in the treatment of female-specific malignancies, which account for more than 60% of cancer cases among women. Therefore, elucidating the underlying molecular mechanisms is an urgent need in gynecological cancers to foster novel therapeutic approaches. Notably, Notch signaling, including either receptors or ligands, has emerged as a promising candidate given its multifaceted role in almost all of the hallmarks of cancer. Concerning the connection between Notch pathway and drug resistance in the afore-mentioned tumor contexts, several studies focused on the Notch-dependent regulation of the cancer stem cell (CSC) subpopulation or the induction of the epithelial-to-mesenchymal transition (EMT), both features implicated in either intrinsic or acquired resistance. Indeed, the present review provides an up-to-date overview of the published results on Notch signaling and EMT- or CSC-driven drug resistance. Moreover, other drug resistance-related mechanisms are examined such as the involvement of the Notch pathway in drug efflux and tumor microenvironment. Collectively, there is a long way to go before every facet will be fully understood; nevertheless, some small pieces are falling neatly into place. Overall, the main aim of this review is to provide strong evidence in support of Notch signaling inhibition as an effective strategy to evade or reverse resistance in female-specific cancers.

## INTRODUCTION

Cancer is one of the principal causes of death worldwide among women in both high-income and low/middle-income countries^[1]^. Notably, female-specific cancers such as breast, cervix, uterus corpus endometrial, and ovarian cancers (OCs) account for about 60% of cancer cases and deaths among the female population worldwide^[1]^. All these numbers reflect the magnitude of female cancers incidence and highlight how the management of these malignancies is still challenging. This is mainly due to frequent tumor relapses promoted by the resistance to common chemotherapeutic agents or targeted therapies, especially for breast^[2]^ and ovarian^[3]^ cancers.

On the whole, drug resistance can be divided into two wide categories, intrinsic or acquired resistance, depending on the presence of pre-existing resistance-mediating factors or their development during the treatment, respectively^[4]^. Despite this binary categorization, the underlying molecular mechanisms are the same^[5]^ and rely on several factors, such as genetic instability, heterogeneity, enhanced drug efflux, inactivation of the drugs, epithelial-to-mesenchymal transition (EMT), cancer stem cell (CSC) phenotype acquisition, and the involvement of the tumor microenvironment (TME)^[6]^.

Since it has been extensively demonstrated that drug resistance limits the effectiveness of cancer therapy, finding novel therapeutic targets to evade or reverse it becomes of paramount importance^[7]^.

An increasing number of studies has focused on the involvement of Notch signaling in the promotion of drug resistance in female cancers, hence evaluating the efficacy of targeting this pathway, as Notch signaling has demonstrated an important role in the development of normal female-specific tissues as well as in the carcinogenesis and tumor progression of several cancers, including breast, cervical, endometrial, and ovarian cancers^[8,9]^.

**Figure 1:**
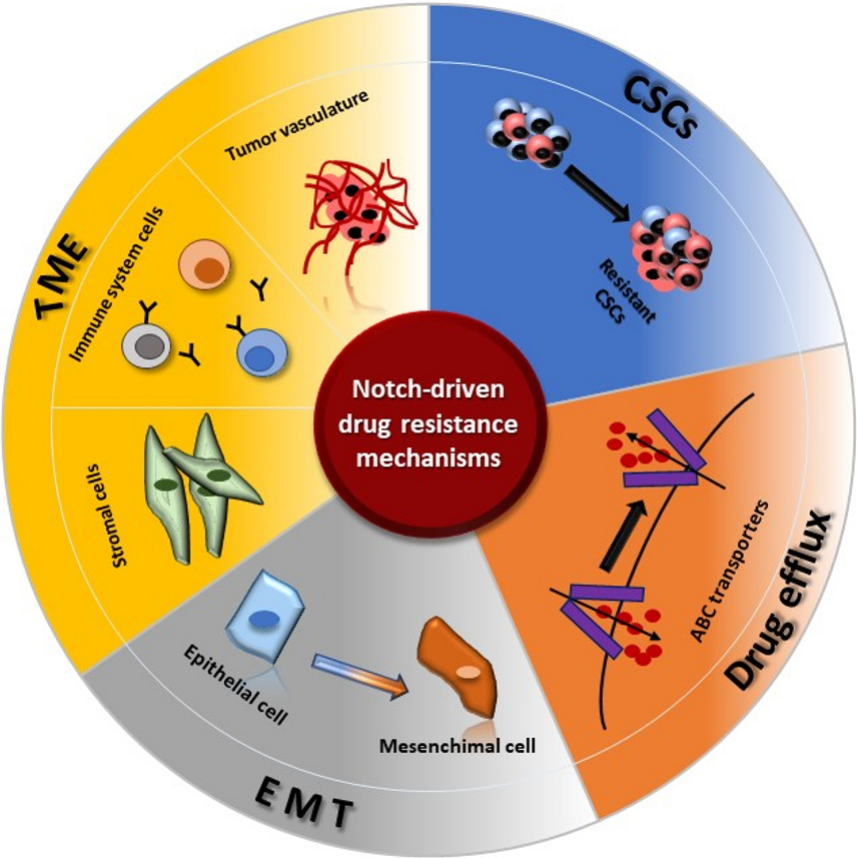
Notch-driven drug resistance mechanisms. The cartoon schematically depicts the involvement of Notch signaling in several drug resistance mechanisms [CSCs (cancer stem cells), drug efflux, EMT (epithelial-to-mesenchymal transition), and TME (tumor microenvironment)].

In the present review, we overview the Notch-dependent molecular mechanisms which drive drug resistance in gynecological cancers (as depicted in Figure 1). On the one hand, CSCs and EMT have been deeply studied and we summarize the literature where Notch targeting overcomes drug resistance by interfering in these processes. On the other hand, we evaluate the role of Notch signaling in the remaining mechanisms even if the connection between Notch targeting and evasion or reversion of resistance has not yet been thoroughly investigated, thus giving hints for further studies.

## OVERVIEW ON THE GYNECOLOGICAL CANCERS

Breast cancer (BC) is the most frequently diagnosed tumor and the principal cause of cancer-related death among women worldwide with the highest BC incidence rates in North America, Australia, New Zealand, and Northern and Western Europe. In 2008, about 1.4 million new cases were estimated worldwide^[10]^ with nearly 459,000 related deaths^[11]^. It has been predicted that the worldwide incidence of this female cancer will reach approximately 3.2 million new cases per year by 2050^[12]^, suggesting that worldwide cancer incidence is on rise.

In high-income countries (HICs), (such as USA, Canada, Brazil, Israel, Australia, and some European countries), the 5-year survival is 85%, while in low/middle-income countries (LMICs) (such as South Africa or India), the survival rate decreases to 60%^[13]^. Furthermore, it is estimated that the probability for a 30-year-old woman to develop BC over a 10-year period is about 10 times less than that for a 70-year-old one^[14]^. The prevalence of BC in the young population is increasing. In addition, in the younger population, the tumor is more aggressive^[15]^, showing a greater rate of recurrence than in older women^[16]^.

BCs are divided into five subtypes based on the expression of estrogen receptors (ER), progesterone receptors (PR), and HER2 oncogene. Overall, ER-positive tumors are usually smaller and lower grade than ER-negative ones^[17]^. The lack of expression of ER, PR, and HER2 characterizes the triple-negative breast cancer (TNBC) subtype, which accounts for approximately 15% of all BC cases^[18]^, and it is the most aggressive subtype with the poorer outcomes^[19]^. TNBC incidence is generally higher in younger women, African and American women, and in patients with mutated BRCA1 gene^[20]^.

According to site, BCs are distinguished in non-invasive and invasive, also recognized as “metastatic” BC^[21]^.

In general, the BC risk factors include genetic history of the disease, BRCA1 or -2 mutations, endogenous estrogen, exposure to drinking, sedentariness, and the use of exogenous hormones^[22]^.

The primary option of BC treatment remains surgical intervention^[23]^. However, several broad classes of drugs are chosen according to tumor molecular characteristics: (1) systemic chemotherapy, which is generally recommended after definitive surgery; (2) hormonal treatment, such as anti-estrogen drugs; or (3) targeted therapies, which include the use of monoclonal antibodies. The most common drugs used for chemotherapy are docetaxel, paclitaxel, platinum agents (cisplatin and carboplatin), vinorelbine (Navelbine), capecitabine (Xeloda), liposomal doxorubicin (Doxil), and cyclophosphamide (Cytoxan)^[24]^. In addition, radiation therapy is used in combination with surgical intervention to optimize the treatment for each person’s anatomy and reduce acute or long-term toxicity^[25]^. Current research efforts are oriented towards more personalized treatments to minimize side effects and improve patients’ survival^[26]^.

OC is the second most common malignancy in women over the age of 40, after BC^[27]^. Every year, about 200,000 new OC cases and 150,000 deaths are estimated worldwide^[28]^, thus representing the fifth leading cause of cancer-related death in women^[29]^.

The high death-to-incidence ratio is mainly due to the absence of specific symptoms and effective screening strategies; as a result, OC is diagnosed at an advanced stage of the disease, when metastases are distributed in the abdomen^[30]^. The incidence rates are lowest in Asia and Africa and highest in Northern and Eastern Europe, where there are also the highest mortality rates^[31]^. OC is rare in young women, especially under the age of 30. Conversely, the risk of incidence increases with age, with a drastic increase after the age of 50^[31]^.

OC is classified into three main subtypes: epithelial, germ cell, and stromal. Among the epithelial group, the most common histological type is the serous carcinoma, followed by endometrioid, clear cell, and mucinous histotypes^[32]^.

It is reported that reproductive factors, hormonal factors, and lifestyle factors are associated with the risk of OC incidence^[3,33]^.

OC-bearing patients are primarily undergoing a standard care consisting of combined cytoreductive surgery followed by platinum- and taxane-based chemotherapy^[34]^. The risk of relapse is around 50% within two years, mainly attributed to chemotherapy resistance^[3]^.

Cervical cancer (CC) is the fourth most frequently diagnosed cancer and represents a major global health challenge^[35]^. It is the fourth leading cause of cancer-related death^[36] ^with an overall survival estimated between 60% and 70%^[13]^, showing a reduction by more than half over the past 30 years, thanks to the introduction of screening programs^[37]^. In LMICs, where mortality is 18 times higher than that observed in developed countries, it is the third most common cause of cancer death^[31]^.

The median age at diagnosis is 47 years in the United States, where almost 50% of cases are diagnosed under age 35 years^[38]^. In South Africa, more than 25% of diagnoses are in women aged 40-49 years between 2004 and 2012^[39]^.

The main risk factor for this type of cancer is chronic infection with human papillomavirus (HPV)^[40]^. It has been estimated that approximately 291 million women have a cervical HPV infection at any given time^[41]^. However, about 85% of infections are spontaneously cleared from the body within a few years and only the persistent infections constitute a risk for cancer development^[42]^. To date, CC may be considered nearly completely preventable thanks to the availability of HPV vaccine and screening programs^[1]^, which also allows the diagnosis in early stages of tumors.

Cervical pre-cancerous lesions can be detected and treated early with cryo-therapy, loop electrosurgical excision procedure, or thermo-coagulation^[43]^, while the treatment of cancer lesions follows the criteria of the International Federation of Gynecology and Obstetrics clinical staging and derives from the disease extension at diagnosis. It might involve radical hysterectomy, chemo-radiation, or their combination^[44]^. Nevertheless, the use of the anti-vascular endothelial growth factor (VEGF) agent bevacizumab, which impinges on the vascularization phase of cancer cells required for tumor survival, has been shown to be capable of extending the overall survival beyond 12 months, and it is currently used in combination with carboplatin and paclitaxel in cancer treatment^[45]^.

Cancers of the uterine corpus, among which endometrial cancers (EC) are the most frequent, account for about 5% of worldwide cancer incidence among women. Incidence rates are generally higher in HICs, where early diagnosis and treatment is common due to early symptoms and the 5-year survival is around 80%^[46]^. However, the cancer survival is lower in LMICs due to the few health services and treatments available^[47]^. The risk factors for uterine corpus cancer include excess body weight or diabetes, estrogen therapy, early menarche and late menopause, and polycystic ovary syndrome^[48]^. In contrast, pregnancy, oral contraceptive use, physical activity, and, unlike other cancers, smoking seem to be protective against risk^[49]^. The primary options for EC-bearing patients are surgery, radiotherapy with or without chemotherapy, or chemotherapy alone^[50]^.

As mentioned above, drug resistance is mainly responsible for causing treatment failure in almost all cancers, including the female-specific ones^[51]^.

To date, the major hope in the fight against those aggressive cancers is the discovery of novel “druggable” targets to evade or reverse resistance, and Notch signaling represents a promising candidate.

## NOTCH STRUCTURE AND SIGNALING AT A GLANCE

**Figure 2:**
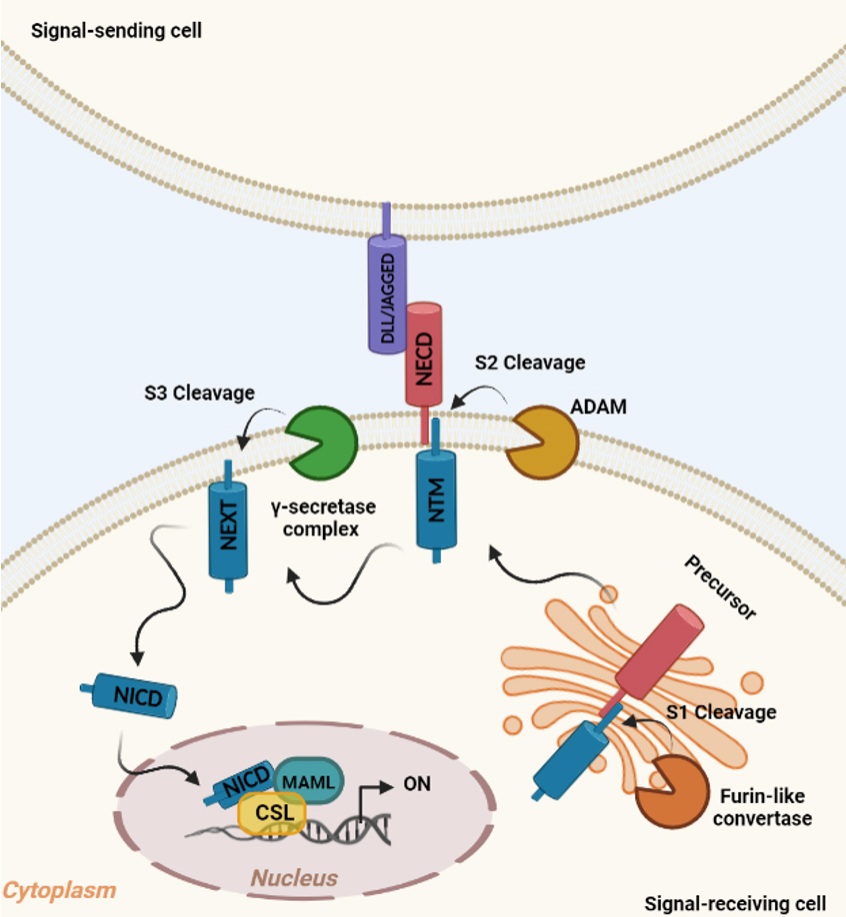
The canonical Notch signaling pathway. In the signal-receiving cell, the Notch receptor precursor is cleaved (S1) by Furin-like convertase in the Golgi compartment, thereby producing Notch extracellular domain (NECD) and Notch transmembrane region (NTM). Upon ligand binding (DLL/JAGGED) expressed on signal-sending cell, NTM is cleaved (S2) by ADAM, resulting in Notch extracellular truncated intermediate (NEXT) exposition to γ-secretase complex cleavage (S3). S3 cleavage allows the release of Notch intracellular domain (NICD), which translocates to the nucleus and interacts with transcriptional regulators (MAML and CSL) to activate the Notch target genes (ON).

The conserved Notch signaling pathway acts as a mediator of short-range cell-cell communication between neighboring cells and controls the cell proliferation, differentiation, and apoptosis^[52]^. As depicted in Figure 2, Notch receptors are single pass transmembrane proteins, initially synthesized as inactive precursors in the endoplasmic reticulum and subsequently cleaved by a furin-like protein convertase in the Golgi compartment^[53]^. The first cleavage (S1) produces heterodimers expressed on the plasmatic membrane and containing a N-terminal ligand-accessible Notch extracellular domain (NECD) and a C-terminal Notch transmembrane region (NTM)^[54]^.

The canonical Notch signaling is triggered by the association to specific ligands that mediate the interaction with adjacent cells^[55]^. Mammals have four Notch paralogs (Notch1-4) with variable structural homology that display both redundant and unique functions^[56]^ and five canonical Notch ligands: Delta-like family (Dll1, Dll3, and Dll4) and Jagged family (Jagged1 and Jagged2)^[57]^. Following the binding to the Notch ligand, an S2 cleavage is triggered by the metalloprotease ADAM leading to the dissociation of the NECD and the production of a membrane-associated Notch extracellular truncated (NEXT) intermediate^[58]^ further cleaved (S3) by a γ-secretases enzyme complex (Presenilin 1 and 2, Nicastrin, APH1, and PEN2)^[58,59]^. The S3 cleavage results in the release of the active Notch intracellular domain (NICD) from the membrane, thus free to transfer to the nucleus where it interacts with the CBF1, suppressor of hairless, and Lag-1 (CSL) families of DNA binding proteins^[60]^. Subsequently, it forms an active complex with the co-activator mastermind-like family (MAML) leading to the upregulation of downstream target genes, such as hairy and enhancer of split (HES)^[61]^. In the absence of NICD, CSL interacts with a co-repressor complex, thus suppressing the transcription [Figure 2]^[62]^.

The Notch signaling pathway is known for its role in the regulation of cell self-renewal, differentiation, and proliferation during development^[63]^, as well as in the maintenance of homeostasis in adult tissue^[64]^. Not surprisingly, given its pivotal role in several processes, dysregulation of Notch signaling has been implicated in the development of cancer, as either an oncogene or a tumor suppressor^[65]^. Over the years, an onco-suppressive action of Notch receptors has been described in basal cell carcinoma of the skin, hepatocellular carcinoma, and B-cell lymphoblastic leukemia^[66,67]^. Conversely, Notch signaling acts as an oncogene in T-cell acute lymphoblastic leukemia where there are mutations affecting Notch1 in more than 50% of cases^[68]^. Notably, several studies documented the oncogenic role of Notch receptors in female-specific cancers^[9]^. In particular, dysregulation of Notch signaling is involved in the promotion of BC^[69]^, as well as in several hallmarks of OC such as proliferation, apoptosis, and metastasis^[70]^. Furthermore, the upregulation of the Notch pathway is a frequent event in EC, where it can increase the invasiveness of tumor cells^[71]^, and it is also associated with malignant behavior and poor prognosis in CC^[72]^.

Overall, the numerous reports regarding Notch signaling and gynecological cancers suggest the importance of considering it as a therapeutic target, especially in terms of drug resistance, as described in the following sections.

## NOTCH SIGNALING AND CSCs

CSCs are a small subset of cancer cells with self-renewal potential, capable of giving rise to a heterogeneous tumor population^[73,74]^. CSCs are characterized by specific features, including the intense tumorigenic potential, the ability to grow as spheres under serum deprivation, and high levels of aldehyde dehydrogenase (ALDH1) activity^[75]^. Many studies have demonstrated that CSCs are more resistant to chemotherapy because of the higher expression of the anti-apoptotic proteins and multidrug resistance genes^[76]^. Indeed, during primary chemotherapeutic treatment, CSCs play an important role in tumor recurrence, preserving tumor growth and heterogeneity through various drug resistance mechanisms^[77,78]^ involving ALDH1 activity, DNA repair, and the activation of pro-proliferative signaling pathways ^[79]^. Notably, the acquisition of stem cell characteristics is also linked to EMT^[80]^, further adding another molecular mechanism for CSC-dependent drug resistance. Indeed, the concept of CSCs displaying both EMT and self-renewal properties provides the rationale for cancer cells to migrate and populate metastatic sites. Part of this expansion is due to an influence imposed by CSCs on non-CSCs to recall an EMT program in the cells, thus shifting them toward drug-resistant CSCs^[75]^.

The role of Notch signaling in CSC functions has been well defined in several tumors^[81]^. Specifically, Notch pathway dysregulation is involved in the acquisition and maintenance of CSC-like properties by sustaining their self-renewal capacity^[67]^. As described in the following section, Notch pathway is also responsible for inducing EMT, which may result in the transformation of epithelial-like CSCs into cells with aggressive mesenchymal-like phenotypes^[82]^, thereby highlighting the potentiality of inhibiting Notch to hamper the interplay between CSCs and EMT phenomena.

Furthermore, a vast body of literature correlates Notch pathway to CSC-driven chemo-resistance^[83,84]^.

Exposure to chemotherapy, such as doxorubicin or docetaxel, as well as a hormone-based therapy, such as tamoxifen or fulvestrant, leads to an enrichment of ALDH+ BC stem cells (BCSCs) displaying resistance to these treatments^[85]^. Notch signaling is known to be pivotal for the maintenance of BCSCs and highly correlated with drug resistance^[86,87]^. Indeed, several studies documented that blocking Notch signaling reduces the stem-like population of BC cells by preventing mammosphere formation^[88,89]^. For instance, inhibition of Notch1 via the bioactive compound psoralidin or Notch1 silencing blocked the growth of ALDH1+ cells, thus resulting in a low mammosphere formation, increased apoptosis, and limited tumor growth in mice models^[90]^. Regarding chemotherapy, it has been demonstrated that TNBC cells treated with gemcitabine or paclitaxel displayed high levels of hypoxia-inducible factors (HIFs) activity, which is correlated with an increased BCSC population^[91]^. Hypoxia exploits Notch signaling pathways to regulate the development of CSCs^[92]^. In this scenario, Yan *et al.*^[93]^ observed that Notch and Wnt signaling pathways may be activated by HIF-2α overexpression, under hypoxia conditions, thus leading to the stem cell phenotype conversion in BCSCs and the overexpression of BCSC markers associated with paclitaxel resistance.

JAK/STAT pathway is crucial for BCSC self-renewal and cancer chemoresistance^[94]^. In addition, EZH2 binds to STAT3, which leads to the enhanced STAT3 activity via its increased tyrosine phosphorylation^[95]^. In keeping with these findings, more recently, it has been documented that chemotherapy activates the EZH2/STAT3 pathway in tumor cells, causing an increase in miR-378a-3p and miR-378d levels, in both cells and exosomes, which finally target the Notch pathway suppressor NUMB. This resulted in Notch overexpression and positive regulation of BCSC markers expression, thus confirming the involvement of Notch stem cell-associated pathways with paclitaxel and doxorubicin resistance of TNBCs^[96]^.

Moreover, Qiu *et al.*^[97]^ demonstrated that docetaxel treatment results in increased primary mammosphere formation and the specific block of Notch1 signaling enhanced chemotherapy efficacy by targeting BCSCs *in vitro* and in patient-derived xenograft (PDX) breast cancer models.

It is worth mentioning that a molecular regulation mechanism of both Notch1 and Notch4 proteins has been shown, through which it is possible to control the BCSC drug resistance. In particular, Rustighi* et al.*^[98]^ showed that both receptors are able to escape from the Fbxw7α-dependent proteasomal degradation following interaction with the prolyl-isomerase Pin1, which is required for the Notch-dependent induction and maintenance of stem cell self-renewal in BSCSs. Furthermore, ablation of Pin1 reduced the expression levels of Notch1/4, thus eliciting sensitivity to chemotherapeutic drugs and inhibiting tumor growth and metastatic spread *in vivo*^[98]^.

Concerning hormone-based therapy, Notch4 and its ligands have been demonstrated to play a crucial role, as Notch4 is found upregulated in BCSCs and conferred resistance to tamoxifen in part through the sustainment of BCSC amplification^[99]^. Additionally, Jagged1-Notch4 signaling activation in ALDH1+ cell populations has been observed as a determining factor in the acquisition of endocrine resistance in patient-derived BCSCs. Consequently, the inhibition of Notch4 via γ-secretase inhibitors (GSIs) *in vivo* is able to abrogate BCSC activity, thus decreasing mammosphere formation of BC isolated cells in acquired tamoxifen resistant PDX tumors^[100]^. In keeping with these findings, more recently, it has been reported that FK506-binding protein-like (FKBPL) reduces endocrine therapy BCSC-mediated resistance through the downregulation of Notch4 and its ligand Dll4^[101]^.

Several studies documented that cancer cells gain resistance to targeted therapies by upregulating compensatory signaling pathways, including Notch signaling. As a result, Notch inhibition represents a promising approach to restore sensitivity to targeted treatments^[102,103]^.

For instance, it has been demonstrated that Notch3-specific inhibition increases TNBC sensitivity to the tyrosine kinase inhibitor (TKI) gefitinib, in TNBC-resistant cells, through regulating EGFR localization, thus rendering it readily targetable by the TKI gefitinib^[102]^. Moreover, a Notch binding sequence has been reported within the HER2 promoter, thus suggesting a mechanism for Notch/HER2 crosstalk^[104,105]^. HER2 expression is shown to be dependent upon Notch signaling in BCSCs: Farnie *et al.*^[106]^ obtained promising results by combining therapies targeting Notch and HER2, respectively, in ductal carcinoma *in situ* stem cells. Furthermore, blocking Notch activity by GSIs induces the downregulation of HER2 protein expression, as well as at the mRNA level, in HER2+ BC-derived mammospheres^[107]^. These studies documented the pivotal interaction between Notch1 and HER2 pathways, both of which are involved in the progression of breast cancer and regulation of BCSCs.

In keeping with these results, upon lapatinib treatment, which targets both EGFR and HER2 receptors, it has been observed that HER2+ breast cancer cells are enriched of high membrane-Jagged1-expressing BCSCs, thus resulting in a higher mammosphere forming efficiency causing lapatinib resistance^[108]^. In addition, the expression of Notch1, Jagged1, and their targets are increased after treatment with trastuzumab, which is a monoclonal antibody against HER2. The combined downregulation of Notch1 sensitizes BC cells to trastuzumab treatment^[109]^. More recently, Baker *et al.*^[110]^ showed that Notch1 contributes to sustain the trastuzumab resistance of HER2+ BC cells, by influencing the survival and tumor-initiating potential of BCSCs through the repression of PTEN, which results in the activation of the pro-proliferative ERK1/2 signaling. These findings suggest that the high expression of Notch1 may predict poorer survival in women with trastuzumab-resistant BC^[110]^. Moreover, PTEN downregulation increases the PI3K/Akt activity, frequently hyperactivated in TNBC^[111]^. The PI3K/Akt/mTOR signaling pathway is also imperative for the regulation of CSC self-renewal, and the cooperation of Notch and PI3K/Akt/mTOR signaling contributes to induce tumorigenesis and chemoresistance in solid tumors^[112,113]^. Similarly, highly expressed activated Akt is associated with chemoresistance in BC^[114]^ while PI3K/mTOR inhibition sensitizes resistant cells to cytotoxic agents^[115]^. These findings suggest that inhibition of Notch sensitizes BC cells to chemotherapy by upregulating PTEN and consequently dampening PI3K/Akt/mTOR signaling.

It has been also demonstrated that Notch-dependent CSCs population sustains the resistance to PI3K or TORC1/2 inhibitors treatment in TNBCs. Indeed, Notch1 activity is induced in TNBC cells by the use of a PI3K or TORC1/2, inhibitor and this is associated with the upregulation of mitochondrial metabolism and FGFR1 signaling. In particular, genetic blockade of Notch1 combined with PI3K or TORC1/2 inhibition abrogates the increase of BCSC markers, mammosphere formation, and *in vivo *tumor-initiating capacity, thus eradicating drug-resistant BCSCs^[116]^.

Furthermore, Notch1-mediated crosstalk with the transcription factor NF-κB signaling is evident in different tumors and both pathways are essential in maintaining the survival of CSCs^[117]^. An example of this interplay is explained in cervical and breast cancers where Notch1 forms a complex with the kinase IKK, which represents the core element of the NF-κB cascade, thus allowing its nuclear translocation^[118,119]^. However, the full interaction between these two pathways in the context of CSCs is not well understood. Recently, Hossain *et al.*^[120]^ demonstrated the involvement of Notch1 in the NF-κB activation pathway and Akt phosphorylation via IKK kinase in TNBC cell resistance and CSCs formation. Pharmacological inhibition of Notch cleavage by GSI (PF-03084014) in combination with Akt inhibitor (MK-2206) or IKK inhibitor (Bay11-7082) blocks mammosphere formation and drug resistance, thus suggesting that combination strategies targeting at the same time Notch and PI3K/Akt or IKK/NF-κB may have potential therapeutic applications in targeting CSCs in TNBC context^[120]^.

The above-mentioned preclinical data clearly demonstrate that Notch signaling is crucial for BC drug resistance and provide evidence of the ability of Notch inhibitors to sensitize cells, including BCSCs, to treatments. Concerning clinical data, interesting results were obtained mainly in TNBCs. Two recent phase I clinical trials have deployed the use of GSIs in combination with chemotherapy for the treatment of patients with advanced BC, including TNBC subtype^[121]^. A combined effect of Notch GSI inhibitors (PF-03084014) and docetaxel was recently observed in a phase 1b study (NCT01876251), thereby showing clinical benefit in 25 patients with advanced TNBC^[122]^. Furthermore, this combination decreased ALDH1+ subpopulations and abrogated BCSCs by targeting the Notch signaling pathway, thus resulting in the reversion of drug resistance^[123]^. Moreover, Schott *et al.*^[124]^ showed that residual BCSC subpopulation is insensitive to docetaxel alone. However, in tumor-derived xenografts, GSI treatment (MK-0752), together with docetaxel, by affecting both NICD and HES1 expression, results in reduced BCSC population. In this study, the authors also included 30 patients with relapsed BC disease after treatment with anthracyclines (NCT00645333) and demonstrated that multiples cycles of GSI treatments produces a reduction in ALDH+ BC cells, as well as a partial response in 11 patients, finally suggesting that additional treatment cycles are required to amplify BCSC reduction and tumor burden^[124]^.

In addition to GSI treatments, other approaches to inhibit Notch signaling have also been evaluated in clinical trials. Preliminary activity against Notch has been further demonstrated by using tarextumab, a first-class anti-Notch2/3 antibody, in a recently completed phase I clinical trial for the treatment of advanced solid tumors (NCT01277146), including breast cancer. Gene expression assays confirmed the downregulation of BCSC markers along with Notch2 and Notch3 inhibition^[125]^, thus suggesting that tarextumab may be used in combination with other therapies that eradicate CSC population.

Both Notch1 and Notch3 are also involved in the self-renewal and maintenance of the CSC properties in OC (OCSCs)^[126,127]^. It has been shown that the expression of Notch1 is strongly associated with the expression of ALDH1, which in turn increased the proliferation of OCSCs and their spheroid formation^[128]^. Interestingly, the combined inhibition of Notch1 and ALDH1 obtained by using liposomal doxorubicin DOXIL® and the anticancer compound withaferin A co-treatment produces a significant reduction in the tumorigenic functions of OCSCs. Therefore, the combination treatment elicits synergistic effects targeting OCSCs, thus suggesting an important potential approach to minimize the induction of Notch-dependent platinum resistance and recurrence of OC^[128]^.

In high grade serous OC, a link between Notch3 and ALDH1 has been reported, already recognized as important in tumor formation^[129]^ and drug resistance^[130]^. Interestingly, Kim *et al.*^[131] ^showed that the combined co-expression of Notch3 and ALDH1 observed in human OC tissues correlates with all poor prognosis parameters, advanced clinical stage, and chemo-resistance in OC.

Moreover, Notch inhibition in paclitaxel-resistant OC cells significantly led to a decrease in viability and migration while apoptosis increased. Notably, both Notch3 silencing and pan-Notch inhibition via GSI treatments are able to induce reduced self-renewal ability of the OCSCs, accompanied by a significant downregulation of stem cell markers, including ALDH1, CD44, CD133, and SOX2. Together, these findings demonstrate that Notch3-specific blocking inhibits OCSC activation and re-sensitizes paclitaxel-resistant OC cells to paclitaxel with an efficacy comparable to GSI treatments^[132]^. In keeping with these findings, another study showed that Notch3 expression is regulated by miR-136 in association with paclitaxel resistance. The authors demonstrated that low expression of miR-136 is correlated with poor prognostic clinico-pathological parameters in OC-bearing patients, while its over-expression is associated with a reduction of proliferation, CSC spheroid formation, and CSC markers expression and increased apoptosis of paclitaxel-resistant cells. Mechanistically, miR-136 is able to suppress Notch3 expression by directly binding to its 3-UTR, thus acting as a tumor suppressor. These observations provide evidence that the miR-136-Notch3 signaling axis plays a critical role in the development of OC chemo-resistance, thus suggesting a potential novel therapeutic target for OC treatment^[133]^.

In this regard, McAuliffe *et al.*^[134]^ strongly supported the specific role of Notch3 signaling pathway in OCSC maintenance and tumor resistance to platinum using both *in vitro* and *in vivo *studies. They showed that inhibition of Notch3, by GSI or siRNA transfection, increases tumor sensitivity to platinum therapy but only the cisplatin/GSI combined treatments may be effective in targeting OCSCs, thus being critical for tumor eradication, and finally improving the sensitivity of OC cells to cisplatin response via enhancing the cellular response to DNA damage^[134]^.

The nuclear orphan receptor NR2F6 expression is directly correlated with tumor progression, especially in epithelial ovarian cancer (EOC), but its role remains largely unclear. Consistently, Li *et al.*^[135] ^demonstrated the interaction between NR2F6 and Notch signaling, where it promotes the transcriptional activity of the Notch3 through the interaction with its promoter and the enrichment of histone acetylase p300 on this, thus inducing Notch3 signaling activation, which results in increased OCSC properties and promoting cisplatin resistance^[135]^.

Jiang *et al.*^[136]^ also demonstrated that treatment with the specific GSI DAPT inhibits self-renewal and stemness development of OCSCs by downregulation of Oct4 and Sox2 marker proteins, required for their maintenance^[136]^ and the promotion of EMT^[137]^.

Moreover, it has been observed that the human follicle stimulating hormone (FSH) inhibits apoptosis in OC cells by positively regulating the Oct4 stem cell signaling pathway and induces OCSC expansion and cisplatin resistance through Oct4-mediated Notch upregulation. This suggested that the stimulation of FSH-stem cells is strongly associated to Notch-dependent drug resistance in OC cells^[138]^.

Moreover, to prevent tumor recurrence, it has been observed that the Notch signaling pathway can be influenced by using different compounds, known for their ability to target other receptors involved in different signaling pathways. For instance, Notch can be downregulated by using the inhibitor poziotinib, a pan-human HER inhibitor, which is also able to inhibit the expression of target genes of Notch, such as c-MYC, thereby suppressing OC stem cell growth^[139]^, while it can be strongly activated by galectin-3, a member of the lectin family. Galectin-3, by favoring Notch signaling, is able to regulate stemness of OC cells, spheroids formation, and their mobility, thus emphasizing that the Notch pathway may be considered at the intersection between CSCs and EMT processes, which are crucial in cancer drug resistance acquisition, as well as in cancer cell plasticity.

Interestingly, galectin-3 directly interacted with the intracellular domain of Notch1 (NICD1) and increased its nuclear translocation. Indeed, downregulation of galectin-3 reduced the levels of cleaved NICD1 and the transcription of the Notch target genes, Hes1 and Hey1. In addition, an increased expression of galectin-3 has been observed in OC patients at advanced stages compared to stages 1 or 2, thus suggesting its involvement in supporting stemness and drug resistance by affecting Notch1 cleavage^[140]^.

More recently, Islam *et al.*^[141]^ observed that the use of the natural compound eugenol, through downregulation of Notch and Hes1 expression, can effectively reverse chemo-resistance through the depletion of OCSCs in Notch1-dependent pathway.

Since various findings suggested a close relationship between drug resistance and Jagged1/Notch signaling, Jagged1 has been found to be a possible target to overcome resistance^[142]^. Indeed, downregulation of Jagged1 in the taxane-resistant OC cells increases docetaxel sensitivity of cells. In keeping with these data, Liu *et al.*^[143]^ reported that GATA1, by binding directly to the ligand, represents the upstream regulatory factor controlling Jagged1-Notch dependent cancer stem cell progression and OC metastasis through enhancing migration, invasion, and EMT. These results confirm the role of Jagged1 in enhancing OC stemness, EMT, and drug resistance through activating Notch signaling pathway^[143]^.

As described above, the crosstalk between hypoxia and Notch signaling pathways controls the development and maintenance of CSCs. It has been suggested that the Notch pathway is required for HIF-1α-induced maintenance of CSCs^[92]^. Seo *et al.*^[144]^ reported that the hypoxia/Notch-1/Sox2 signaling is also crucial for maintaining OCSCs and, taken together, can thereby serve as a candidate for novel anti-CSCs therapeutics. Furthermore, the association between hypoxia and Notch signaling promotes drug resistance and self-renewal of OCSCs. Indeed, Notch signaling facilitates hypoxia-induced upregulation of Sox2, which induces drug resistance in CSCs through the upregulation of the ABC transporters, ABCB1 and ABCG2, commonly involved in the efflux of anticancer agents. Consequently, DAPT treatment abolishes hypoxia-mediated induction of OCSCs, thereby reversing drug resistance^[144]^.

CSCs have also been proposed to be responsible for high recurrence rate and chemo-resistance also in cervical carcinoma (CC)^[145]^. Yang *et al.*^[146] ^observed an enrichment of the cervical CSC (CCSC) population and an increase in spheroid formation after cisplatin treatment. Interestingly, CCSCs display the upregulation of Notch expression, suggesting its specific correlation with cisplatin resistance also in CC. Indeed, the doxycycline-dependent inhibition of Notch blocks the proliferation and differentiation rate of CCSCs, bringing an enhancement in cisplatin therapy^[146]^. Moreover, the expression of the stemness markers Oct-4 and Sox-2, Notch1, and the Notch signaling components (ADAM-17, γ-secretase, and JAG1) is found to be significantly elevated in 5-fluorouracil (5-FU)-resistant CC cell lines. Currently, it has been demonstrated that ADAM 17 inhibition through the nano-formulation of Quinacrine (NQC) in 5-FU resistant CSCs leads to the reduction of their proliferation, thus inducing re-sensitization to the treatment^[147]^.

Notably, it has been documented that the Notch pathway is involved not only in CSC-driven chemo-resistance but also in the radio-resistance event. A recent study reported that Notch signaling is upregulated by its interaction with fused toes homolog (FTS) determining the maintenance of CSCs and spheroid formation upon irradiation of CC cells. The authors showed that the silencing of FTS increases radio-sensitivity by blocking Notch1 activation^[148]^.

Concerning endometrial CSCs (ECSCs), it has been demonstrated that they acquire drug resistance due to high expression levels of CD133, a widely accepted biomarker for tumor-initiating cells. Indeed, CD133+ cells exhibited significant resistance to chemotherapy^[149]^. Studies indicated that the activation of Notch signaling pathway is a pivotal event in CD133+ cells as it promotes ECSC cell growth, proliferation, and self-renewal while Notch inactivation can sensitize CD133+ cells to chemotherapy^[150,151]^. Furthermore, the molecular therapeutics targeting key biomarkers such as EGFR receptors showed some success in clinical trials, but the cancer stem cell-derived drug resistance hindered the drug effects^[152]^. Recently, Shang *et al.*^[71] ^found that Notch signaling is expressed in both CD133+ and CD133- cells. However, the activation of Notch signaling in CD133+ cells leads to a higher proliferation rate and lower apoptosis and results in targeted-therapy resistance, compared to CD133- cells. Findings from xenograft experiments further demonstrated that CD133+ cells are able to retain a higher tumorigenic capacity than their counterpart, thus indicating their tumor-initiating feature. In addition, Notch inhibition by DAPT combined with EGFR inhibitor (AG1478) treatment on EC cells displays ameliorative effects compared to DAPT or AG1478 treatments alone, thus reducing the CD133+ cells viability^[71]^.

Therefore, given the pivotal role of Notch signaling in the regulation of CSC activity in drug resistance (as summarized in Table 1), it might be useful to further investigate the regulatory mechanisms of Notch-mediated resistance and evaluate whether inhibition of the Notch signaling cascade can be considered as a potential therapeutic approach to abrogate drug resistance in female cancers.

**Table 1:** Effects of Notch-targeted therapeutics to reverse CSC-driven drug resistance. Summary of the pre-clinical and clinical studies

**Notch-targeted therapeutics**	Target^*^	**Mechanism of action**	**Reverse resistance to**	**Model**	**Cancer context**	**Ref.**
mAb Notch1	Notch1	Decrease of mammosphere formation	Docetaxel (chemo-therapeutic agent)	Preclinical study: mice	Breast	[97]
GSI (γ-secretase inhibitor)	Jagged1/Notch signaling	Decrease of mammosphere formation	Tamoxifen (ER receptor inhibitor)	Preclinical study: mice	Breast	[100]
AD01/ALM201 (FKBPL-based peptide)	Dll4/Notch4	Abrogation of BCSCs activity	Tamoxifen (ER receptor inhibitor)	Preclinical study: mice	Breast	[101]
GSI (γ-secretase inhibitor)	Jagged1/Notch signaling	Decrease of mammosphere formation	Lapatinib (EGFR/HER2 inhibitor)	Preclinical study: cell lines	Breast	[108]
GSI (γ-secretase inhibitor)	Jagged1/Notch1	Downregulation of HER2 expression in mammosphere	Trastuzumab (HER2 inhibitor)	Preclinical study: cell lines	Breast	[109]
shRNA Notch1	Notch1	Downregulation of PTEN expression in BCSCs	Trastuzumab (HER2 inhibitor)	Preclinical study: cell lines	Breast	[110]
GSI-IX (γ-secretase inhibitor)	Notch1	Abrogation of BCSCs activity	BEZ235 + MLN128 (TORC1/2 inhibitor)	Preclinical study: cell lines	Breast	[116]
GSI (PF-03084014)	Notch1	Block of mammosphere formation	MK-2206 (Akt inhibitor) + Bay11-7082) IKK inhibitor	Preclinical study: cell lines	Breast	[120]
Withaferin A (anticancer compound)	Notch1	Reduction of OCSCs function	Doxorubicin (chemo-therapeutic agent)	Preclinical study: mice	Ovarian	[128]
GSI/shRNA Notch3	Notch3	Inhibition of OCSCs activation	Paclitaxel (chemo-therapeutic agent)	Preclinical study: cell lines	Ovarian	[132]
miR-136	Notch3	Abrogate OCSCs activity	Paclitaxel (chemo-therapeutic agent)	Preclinical study: cell lines	Ovarian	[133]
GSI (γ-secretase inhibitor)	Notch3	Abrogate OCSCs maintenance	Cisplatin (chemo-therapeutic agent)	Preclinical study: mice	Ovarian	[134]
shRNA Galectin-3	Notch1	Downregulation of OCSCs stemness	Paclitaxel + Cisplatin (chemo-therapeutic agent)	Preclinical study: cell lines	Ovarian	[140]
Eugenol (natural compound)	Notch1	Depletion of OCSCs	Cisplatin (chemo-therapeutic agent)	Preclinical study: cell lines	Ovarian	[141]
shRNA Jagged1	Jagged1	Downregulation of OCSCs stemness	Docetaxel (chemo-therapeutic agent)	Preclinical study: cell lines	Ovarian	[143]
DAPT (γ-secretase inhibitor)	Notch1	Abolishment of hypoxia-mediated induction of OCSCs	Paclitaxel (chemo-therapeutic agent)	Preclinical study: cell lines	Ovarian	[144]
Doxyclycine (antibiotic)	Notch1	Block of CCSCs proliferation and differentiation rate	Cisplatin (chemo-therapeutic agent)	Preclinical study: mice	Cervical	[146]
NQC (Quinacrine)/GW280264	ADAM17/Notch signaling	Reduction of CCSCs proliferation	5-Fluorouracil (5-FU) (chemo-therapeutic agent)	Preclinical study: cell lines	Cervical	[147]
shFSH	Notch signaling	Abrogation of CCSCs maintenance	Irradiation	Preclinical study: cell lines	Cervical	[148]
DAPT (γ-secretase inhibitor)	Notch signaling	Reduction of ECSC viability	AG1478 (EGFR inhibitor)	Preclinical study: cell lines	Endometrial	[71]
GSI (PF-03084014) (γ-secretase inhibitor)	Notch signaling	Abrogation of BCSCs activity	Docetaxel (chemotherapeutic agent)	Clinical trial: phase Ib (NCT01876251)	Breast	[122]
GSI (γ-secretase inhibitor)	Notch signaling	Reduction of BCSCs proliferation	Anthracyclines (chemo-therapeutic agent)	Clinical trial: phase Ib (NCT00645333)	Breast	[124]
GSI (γ-secretase inhibitor)	Notch signaling	Downregulation of BCSCs markers	Tarextumab (mAb Notch2/3)	Clinical trial: phase Ib (NCT01277146)	Breast	[125]

*Notch receptor or ligand involved, when specified.

## NOTCH SIGNALING AND EMT

The EMT is a process whereby epithelial cells acquire mesenchymal properties through the downregulation of cell adhesion molecules expression (i.e., E-cadherin) and the upregulation of mesenchymal markers expression (i.e., N-cadherin), in order to gain migratory behaviors and invasive properties^[153]^. Increasing numbers of studies have not only suggested that EMT represents an essential process in normal embryonic development but also a significant mechanism involved in the progression of different cancer types^[154]^.

Most importantly, the acquisition of the EMT phenotype is related to chemo-resistance^[155]^, one of the most frequent causes of cancer mortality. Indeed, the targeting of key pathways which can regulate EMT may represent an effective treatment strategy.

Several studies have demonstrated that Notch over-expression is able to induce the loss of E-cadherin, while Notch inhibition increases N-cadherin, thus suggesting that the Notch signaling pathway may represent a crucial regulatory mechanism for EMT^[156]^. Noteworthy, Notch signaling often interacts with other pathways in inducing EMT. Indeed, the authors also reported the presence of a crosstalk between Notch and TGF-β pathway. Particularly, they suggested that Notch could act downstream of the TGF-β signaling, which is known to modulate the invasive and migratory properties of cancer cells. In addition, Kang *et al.*^[157]^ revealed that Notch-1 signaling triggers EMT in different types of cancers, and, conversely, its inactivation is responsible for the EMT suppression.

The two critical EMT-associated markers, Slug and Snail, are regulated by Notch signaling in BC^[158,159]^. Particularly, Slug seems to be a direct target gene of Notch1^[160]^. Consistent with these findings, Notch2 is also able to upregulate some EMT-associated transcriptional regulators, i.e., Vimentin, Twist, Snail, and Slug, in basal type BC cells^[161]^. Jagged1 has been shown to positively regulate Slug via Notch signaling activation, thus resulting in E-cadherin downregulation and EMT promotion^[159,160,162]^. Consistent with a previous report^[93]^, hypoxia-dependent activation of Notch signaling is also correlated with EMT in BC cells, as revealed by E-cadherin and β-catenin downregulation^[158,163]^. Mechanistically, Chen *et al.*^[158]^ revealed that the hypoxia-dependent Notch signaling activation is mediated by both HIF-α isoforms, which synergized with the transcriptional coactivator of Notch receptor, MAML1, in the transcription of Notch target genes.

Increasing evidence suggests that EMT is also associated with drug resistance in BC^[164]^ and that this process is partly responsible for chemo-resistance. Xiao *et al.*^[164]^ demonstrated that Notch1 significantly contributed to chemo-resistance in TNBC cells through the promotion of the expression of major vault protein (MVP), the main component of the vault complex^[165]^. MVP is involved in the export of drugs from the nucleus^[166]^ and is known to confer chemotherapy resistance in various tumor contexts^[167]^. The authors found that the intracellular domain of Notch1 is able to bind the promoter of MVP, thus inducing its transcription, and in turn promotes the AKT pathway activation. The activation of AKT resulted in EMT phenotype as well as in cisplatin and doxorubicin resistance. In the same tumor context, a Chinese study described a positive Notch1 association with the expression of the melanoma cell adhesion molecule (MCAM), an EMT activator protein^[168]^. In particular, the authors observed a time-dependent manner increased expression of both proteins after cisplatin treatment, even higher in cisplatin-resistant TNCB cells. This observation suggests the ability of Notch1 in promoting cisplatin chemo-resistance through MCAM-dependent EMT promotion in TNBCs^[168]^.

In keeping with these findings, Zhang *et al.*^[123]^ demonstrated that combined treatment of TNBC cells with GSI PF-03084014 and docetaxel is able to reverse the EMT phenotype and restore tumor chemo-sensitivity.

NumbL, the Numb homolog implicated in cell adhesion, migration, and division during central nervous system development^[169]^, has been shown to be a tumor suppressor protein in BC also for its negative regulation upon Notch signaling pathway^[170,171]^. In BC cells, the authors observed that NumbL downregulation is correlated with the activation of Notch pathway, further increasing the EMT-dependent transcription of Snail and Twist and inducing chemotherapy resistance. This explains why low expression of NumbL protein is observed in relapsed cancers, including BC^[170]^.

Gu *et al.*^[172]^ investigated Notch3 involvement in adriamycin-resistant MCF-7 BC cells. Notch3 expression is associated with chemo-sensitivity, while its downregulation has been shown to be involved in MCF-7 chemo-resistance. The authors showed that the Fos-related antigen 1 protein, a regulator involved in cell proliferation, differentiation, and transformation and EMT promotion, is negatively regulated by Notch3 in tumor cells, thus revealing the onco-suppressive role of the receptor^[172]^. In keeping with these findings, it has been observed that Notch3 may negatively regulate EMT^[173-176]^, by acting, at least in part, via GATA-3 induction in BC cells^[174]^. This suggest that the involvement of Notch signaling is complex and context-dependent and further investigations are needed.

The role of Notch signaling in EMT promotion is also largely demonstrated in OC. Alterations of Notch pathway are prevalent in OC^[177]^, where the signaling confers metastatic properties by the promotion of EMT, as confirmed by the upregulation of the mesenchymal markers and the downregulation of the epithelial ones^[178]^. Consequently, Notch targeting might represent a promising therapeutic strategy for the chemo-sensitivity restoration in OC context.

It has been demonstrated that thyroid hormone receptor interactor 13 (TRIP13), which is implicated in progression and metastasis of multiple cancers, acts as an oncogene in EOC development by the modulation of Notch signaling^[179]^. In particular, the authors observed that TRIP13 inhibition in OC cells, through the suppression of Notch pathway, finally leads to EMT suppression, thus supporting the Notch involvement in the acquisition of the mesenchymal properties^[179]^. In addition, Bocci *et al.*^[180]^ also showed that the block of the Notch signaling inhibitor Numb can be an effective strategy to modulate Notch-driven EMT.

Some studies investigated the effects of the GSI DAPT on TGF-β-induced EMT, which is found in OC cells but is not expressed in normal ovarian epithelial cells^[181,182]^. DAPT treatment is able to reverse the TGF-β-induced EMT process, thus revealing the TGF-β pathway’s ability to control the OC mesenchymal phenotype via Notch signaling, finally favoring the chemo-resistance acquisition^[181,182]^. These findings contribute evidence that the Notch activation is partially required for the EMT induction by TGF-β also in OC cells.

Notch signaling is also involved in the Rap1A-mediated EMT induction. The Ras-associated protein is able to promote the OC cell proliferation, migration, and invasion by activating Notch pathway, as well as ERK and MAPK pathways promotion and the EMT markers expression^[183]^. Indeed, it has been demonstrated that Notch signaling inhibition is able to revert the expression of those molecules, thus preventing the mesenchymal phenotype.

In cisplatin-resistant OC cells, a Chinese study revealed that Jagged1 is able to interact with the JAK/STAT3 pathway by physically interacting with STAT3 and cooperating with the protein in supporting EMT, further reinforcing the invasion and migration abilities of platinum-resistant OC cells^[184]^. Indeed, the knockdown of Jagged1 reverts EMT both *in vitro* and *in vivo*, thus reducing the cisplatin-resistant cells’ abilities of invasion and migration^[184]^. Furthermore, a recent study reported that Numb knockdown, which in turn affected Notch pathway activation, inhibited cell proliferation, invasion, and migration, thus enhancing the anti-tumor effect of cisplatin in different OC cell lines^[185]^.

Notch3 seems to be specifically involved in the OC EMT-mediated chemo-resistance. Gupta *et al.*^[178]^ demonstrated that Notch3 activation in OVCA429 cells is responsible for the fibroblast-like morphology acquisition, the expression of EMT markers (i.e., Slug and Snail) and the decrease of E-cadherin expression on cell surface^[178]^. Notably, Notch3 expression is able to support OVCA429 resistance to carboplatin by the reduction of chemotherapy-induced apoptosis^[178]^.

The Notch signaling pathway also plays a pivotal role in CC development and progression, and it correlates with invasive and metastatic properties^[186]^. In particular, Wang *et al*.^[72]^ investigated the impact of Notch pathway on the survival, invasiveness, EMT, and chemo-resistance of CC cells; they observed that the expression of Notch2 was higher than in the normal HPV-16-immortalized human cervical epithelial cells CRL2614. Interestingly, the treatment with Notch inhibitor GSI RO4929097 is able to impair not only cancer cells proliferation but also the expression of the mesenchymal markers Snail, Twist, and N-cadherin, thus affecting their migration, invasion, and drug resistance^[72]^.

In CC, radiotherapy represents one of the most common treatments for advanced tumor^[187]^, despite frequently resulting in recurrence. Several studies have demonstrated that the Notch receptor ligand Dll4 could be considered as a prognostic biomarker, thus predicting the pelvic lymph node metastasis in patients with CC^[188]^. In this study, Yang *et al.*^[189]^ observed that the Dll4 expression was higher in radiotherapy-resistant SiHa CC cells in comparison with radiotherapy-sensitive CC Me-180 cells. In addition, they demonstrated that the Dll4 small interfering is able to inhibit the EMT and to reduce proliferation, invasion, and migration of CC cells, thus finally increasing CC radio-sensitivity.

Progestin treatment has been used as a conservative therapy for a long time in EC^[190,191]^, showing recurrence in more than half of treated EC patients^[192]^. For the first time, Zhou *et al.*^[193] ^demonstrated that EMT is also involved in progestin resistance of EC, which represents the main hormone treatment for this cancer. It has been demonstrated that dachshund homolog 1 (DACH1) was a tumor suppressor in progestin-resistant Ishikawa PR EC cells because its overexpression was able to inhibit EMT and reverse drug resistance^[193]^. Interestingly, DACH1 regulated EMT by Notch1 pathway suppression, thus revealing how the Notch pathway is strongly involved in the regulation of EMT also in EC context, being ultimately responsible for therapy resistance.

As highlighted above, the Notch signal pathway can be considered a key regulator in the promotion of EMT, which is in turn involved in drug resistance in various cancer types, including female tumors (as summarized in Table 2). Indeed, its down-modulation may represent a novel approach for cancer treatment in order to overcome not only chemo-resistance but also other types of resistance, such as resistance to hormone-based treatments. However, much has not been investigated and there would be much to learn on this topic.

**Table 2:** Effects of Notch-targeted therapeutics to reverse EMT-driven drug resistance. Summary of the pre-clinical studies

**Notch-targeted therapeutics**	Target^*^	**Mechanism of action**	**Reverse resistance to**	**Model**	**Cancer context**	**Ref.**
shRNA Notch1	Notch1	Inhibition of the major vault protein (MVP)-dependent AKT pathway activation and EMT promotion	Cisplatin + Doxorubicin (chemo-therapeutic agents)	Preclinical study: cell lines	Breast	[164]
shRNA Notch1	Notch1	Inhibition of the melanoma cell adhesion molecule (MCAM)-dependent EMT promotion	Cisplatin (chemo-therapeutic agent)	Preclinical study: cell lines	Breast	[168]
GSI PF-03084014 (γ-secretase inhibitor)	Notch signaling	Inhibition of EMT phenotype	Docetaxel (chemo-therapeutic agent)	Preclinical study: cell lines	Breast	[123]
shRNA Notch3	Notch3	EMT phenotype revertion and enhancement of chemo-therapy-induced apoptosis	Carboplatin (chemo-therapeutic agent)	Preclinical study: cell lines	Ovarian	[178]
shRNA Jagged1	Jagged1	Inhibition of EMT phenotype	Cisplatin (chemo-therapeutic agent)	Preclinical study: mice	Ovarian	[184]
shRNA Numb	Notch signaling	Enhancement of chemo-therapy-induced cell proliferation, invasion and migration	Cisplatin (chemo-therapeutic agent)	Preclinical study: cell lines	Ovarian	[185]
shRNA Dll4	Dll4	Inhibition of EMT phenotype	Irradiation	Preclinical study: cell lines	Cervical	[188]
Dachshund homolog 1 (DACH1) overexpression	Notch1	Inhibition of EMT phenotype	Progestin (endocrine therapy)	Preclinical study: cell lines	Endometrial	[193]

*Notch receptor or ligand involved, when specified.

## NOTCH SIGNALING AND DRUG EFFLUX

The human genome encodes 49 different ATP-binding cassette (ABC) transporters, grouped into seven subfamilies (from A to G)^[194]^. They are responsible for the intracellular levels of small molecules and are involved in physiological processes^[195]^. Notably, ABC transporters such as multidrug resistance protein1 (MDR1/ABCB1), multidrug resistance-associated protein (MRP1/ABCC1), and breast cancer resistance protein (BCRP/ABCG2) are overexpressed in cancer cells^[196]^, and they can efflux anticancer agents, thereby leading to drug resistance^[6]^.

Several studies performed in various tumor contexts indicated that Notch signaling regulates ABC transporter transcription^[197-199]^.

Concerning female-specific malignancies, this relationship was investigated mainly in OC and BC (as summarized in Table 3). For instance, Park *et al.*^[200]^ demonstrated that Notch3 increases the expression of MDR1/ABCB1 in OC cell lines. Indeed, Notch3 knockdown led to the downregulation of MDR1/ABCB1, thus reversing carboplatin resistance^[200]^. Moreover, Zhang *et al.*^[123] ^evaluated the anti-tumor efficacy of the γ-secretase inhibitor PF-03084014 in combination with docetaxel in TNBCs both *in vitro* and *in vivo *studies: the authors demonstrated that PF-03084014 improves taxane-based therapy by multiple mechanisms, among them the decrease of MDR1/ABCB1 and ABCC2 transporters^[123]^.

**Table 3:** Effects of Notch-targeted therapeutics to reverse drug efflux-dependent drug resistance. Summary of the pre-clinical studies

**Notch-targeted therapeutics**	Target^*^	**Mechanism of action**	**Reverse resistance to**	**Model**	**Cancer context**	**Ref.**
PF-03084014 (γ-secretase inhibitor)	Notch signaling	Downregulation of MDR1/ABCB1 and ABCC2 transporters	Docetaxel (chemo-therapeutic agent)	Preclinical study: mice	Breast	[123]
shRNA Notch3	Notch3	Downregulation of MDR1/ABCB1 transporter	Carboplatin (chemo-therapeutic agent)	Preclinical study: cell lines	Ovarian	[200]
lncRNA MALAT1	Notch1	Downregulation of MRP1/ABCC1 transporter	Cisplatin (chemo-therapeutic agent)	Preclinical study: mice	Ovarian	[204]

*Notch receptor or ligand involved, when specified.

Furthermore, ectopic expression of the intracellular domain of Notch1 (N1ICD) correlated to the increase of another ABC transporter (MRP1/ABCC1), both at mRNA and protein levels, in BC cell lines. The authors clearly demonstrated that N1ICD induces the transcription of MRP1/ABCC1 by interacting with CBF1 and its effects in promoting drug resistance by the induction of this transporter^[201]^. Consistent with these findings, in a recent study, Zhang *et al.*^[202]^ demonstrated that the inhibitor of DNA-binding 4 (ID-4) protein sustains chemo-resistance in BC because it is positively associated with Notch1 pathway by favoring CBF1-MRP1/ABCC1. Moreover, Kim *et al.*^[203] ^documented that Notch1 and MRP1/ABCC1 are upregulated after chemotherapy and their protein expression is positively correlated in BC clinical samples. Interestingly, a more recent study documented that Notch1 is also connected to MRP1/ABCC1 in OC, and it confers cisplatin resistance to OC cell lines^[204]^.

Given that it has been widely documented that ABC transporters are highly expressed in CC^[205,206]^ and EC^[207]^, it may be worthwhile investigating whether blocking ABC transporter function by Notch signaling inhibition could represent a potential approach to CC and EC treatment.

## NOTCH SIGNALING AND TME

The extracellular environment where cancer cells reside is generally characterized by hypoxia and low pH^[208]^ and consists of several cellular elements [i.e., cancer-associated fibroblasts (CAFs), immune cells including myeloid-derived suppressor cells (MDSCs), tumor-associated macrophages (TAMs), ECs, *etc.*] and non-cellular ones [i.e., cytokines, chemokines, extracellular matrix (ECM), *etc*.], collectively known as the TME^[209]^.

Notably, given that TME is a dynamic and often-changing network which influences and favors cancer cells, more and more studies are investigating how the TME is involved in responsiveness or resistance to various drugs^[210]^. For instance, stromal cells in the TME interact directly with cancer cells, sustaining their growth and promoting hypoxia, acidosis, and oxidative stress, thus contributing to ECM remodeling to induce angiogenesis and mechanical stiffness^[211]^. Moreover, MDSCs and TAMs protect cancer cells from immune elimination^[212,213]^, and CAFs induce drug resistance by secreting cytokines, chemokines, and exosomes^[214]^. Therefore, since the TME plays a crucial role in cancer resistance to therapies, deepening the understanding of the underlying molecular mechanisms is a promising strategy to overcome drug resistance^[215]^.

In this scenario, Notch receptors are involved in every aspect of the TME^[216,217]^. In the following subsections, we describe their role in shaping the female cancer TME and the consequences of targeting them in a therapeutic perspective (as summarized in Table 4).

**Table 4:** Effects of Notch-targeted therapeutics to reverse TME-driven drug resistance. Summary of the pre-clinical and clinical studies

**Notch-targeted therapeutics**	Target^*^	**Mechanism of action**	**Reverse resistance to**	**Model**	**Cancer context**	**Ref.**
**Tumor-stromal cell interaction**						
15D11 (mAb α-Jagged1)	Jagged1/Notch signaling	Reduction of bone metastasis	Paclitaxel (chemo-therapeutic agent)	Preclinical study: mice	Breast	[221]
DAPT (γ-secretase inhibitor)	Jagged1/Notch3	Inhibition of the expansion of radiotherapy-resistant BCSCs (CD44^+^CD24^low+^)	Irradiation	Preclinical study: mice	Breast	[227]
shRNA Notch3	Notch3	Inhibition of the expansion of hormonal therapy-resistant BCSCs (CD133^high^)	Fulvestrant (ER inhibitor)	Preclinical study: cell lines	Breast	[228]
GSI (γ-secretase inhibitor)	Jagged1/Notch3	Inhibition of the expansion of chemo-resistant OC cells	Cisplatin + Taxol (chemo-therapeutic agents)	Preclinical study: cell lines	Ovarian	[222]
**Tumor-immune cells interaction**						
Ginsenoside Rg3 (natural compound)	Notch signaling	Inhibition of MDSC-induced cancer stemness and EMT	Irradiation	Preclinical study: cell lines	Breast	[244]
RO4929097 (γ-secretase inhibitor)	Jagged1/Notch signaling	Inhibition of tumor-associated macrophages M2 polarization	Aromatase inhibitor (ER inhibitor)	Preclinical study: cell lines	Breast	[245]
**Tumor vasculature**						
ABT-165 (bsAb α-Dll4/VEGF)	Dll4	Disruption of functional tumor vasculature	Paclitaxel (chemo-therapeutic agent)	Preclinical study: mice	Breast	[259]
HB-32 (bsAb α-Dll4/VEGF)	Dll4	Disruption of functional tumor vasculature	Docetaxel (chemo-therapeutic agent)	Preclinical study: mice	Breast	[260]
REGN421 (mAb α-Dll4)	Dll4	Disruption of functional tumor vasculature	Aflibercept (VEGF inhibitor)	Preclinical study: mice	Ovarian	[262]
REGN421 (mAb α-Dll4)	Dll4	Disruption of functional tumor vasculature	Aflibercept (VEGF inhibitor)	Preclinical study: mice	Ovarian	[263]
Endostar (recombinant human endostatin)	Dll4	Restoration of vascular homeostasis	Cisplatin + Paclitaxel (chemo-therapeutic agents)	Clinical study	Cervical	[266]
Demcizumab (mAb α-Dll4)	Dll4	Disruption of functional tumor vasculature	Paclitaxel (chemo-therapeutic agent)	Clinical trial: phase Ib (NCT01952249)	Ovarian	[267]
Navicixumab (bsAb α-Dll4/VEGF)	Dll4	Disruption of functional tumor vasculature	Paclitaxel (chemo-therapeutic agent)	Clinical trial: phase Ib (NCT03030287)	Ovarian	[268]

*Notch receptor or ligand, when specified.

### Disrupting Notch-dependent tumor and stromal cell interactions to overcome EMT- or CSC-driven drug resistance

During tumor progression, it is well known that tumors co-evolve with the surrounding microenvironment^[218]^ and cancer cells recruit stromal cells, which in turn promote drug resistance and metastasis^[219]^.

Among female-specific cancers, the Notch-mediated interaction between cancer and stromal cells has principally been studied in the BC context.

Xing *et al.*^[220]^ found that BC brain metastatic cells secrete IL-1β to activate astrocytes via the NF-κB pathway. Consequently, cancer-activated astrocytes express Jagged1, which interacts with Notch1 in cancer cells to sustain CSCs in brain metastasis^[220]^. In line with these findings, Zheng *et al.*^[221]^ observed that chemotherapy induces the over-expression of Jagged1 in the osteoblasts, thereby leading to the activation of Notch signaling in tumor cells. The authors developed a fully human monoclonal antibody against Jagged1, named 15D11, to disrupt osteoblasts-tumor cells interaction and this was functional to reverse chemo-resistance^[221]^. The role of Jagged1 in activating Notch signaling was also studied in OC: it has been shown that endothelial cells activate Notch3 signaling in OC cells via Jagged1, and this interaction mediates chemo-resistance by sustaining PI3K/Akt and ERK pathways^[222]^.

Furthermore, some studies focused on the Notch pathway and CAFs crosstalk in BC. Overall, on the one hand, Notch signaling is involved in regulating CAFs activation^[223]^, but, on the other hand, CAFs may activate Notch pathway in cancer cells leading to CSCs self-renewal and EMT^[217]^, thereby potentially favoring drug resistance, as described in the previous sections. Concerning the latter phenomenon, Tsuyada *et al.*^[224]^ documented a crosstalk circuit that involves STAT3 and Notch pathway. They showed that STAT3 binds the promoter of the cytokine CCL2 in CAFs, thereby augmenting the secretion of CCL2, which in turn promotes Notch1-dependent breast CSC self-renewal^[224]^. Moreover, it has been demonstrated that CAFs secrete the cytokine IL-6, which enhances Notch3 signaling and consequently invasiveness in ERα-positive BC models. Indeed, IL-6 exposure leads to the chronic STAT3 phosphorylation, which induces the transcription of Notch3 receptor^[225]^. In both cases, CAFs act in a paracrine manner, but they can act also via cell-cell interactions: in particular, Pelon *et al.*^[226] ^reported that Notch1, Notch2, and Notch3 are upregulated in a specific subset of CAFs which promote BC cell invasion in a 3D model.

Interestingly, two studies reported that CAFs promote the upregulation of Notch3 signaling followed by the expansion of therapy resistant breast CSCs through either Jagged1-Notch3 interaction^[227]^ or CAF-derived microvesicles^[227,228]^, thus pinpointing that Notch signaling may be targeted to overcome stroma-mediated resistance in BC. Notably, Boelens *et al.*^[227]^ observed that the paracrine activation of STAT1 increases the transcription of Notch target genes, thus suggesting that anti-Notch-based therapies may be hindered by the activation of different pathways which converge on the same substrates.

These observations highlight the potential benefits of investigating the Notch-mediated crosstalk between tumor cells and the stroma not only in BC context but also in other female malignancies since recent findings underpin the emerging role of stromal cells in drug resistance also in OC^[229,230]^, EC^[231]^, and CC^[232]^.

### Disrupting Notch-dependent tumor and immune cells interaction to overcome drug resistance

It is well established that the interplay between tumor and immune cells in the TME influences immune surveillance and responsiveness to therapies^[233]^.

Notably, Notch signaling is a key regulator of the immune infiltrate within the TME^[234]^. Indeed, both myeloid and lymphoid lineages are affected by Notch pathway^[235,236]^. On the one hand, Notch signaling is involved in the thymic development of T cells^[237]^ and plays a pivotal role in CD8+ T-cell activation and effector functions^[238,239]^. On the other hand, Notch signaling is required for the expansion and activity of immunosuppressive cells, such as MDSCs^[240]^ and TAMs^[241]^. These contradictory findings imply that the function of Notch receptors in tumor immunity is dependent upon components of the TME.

Beyond its function in several subpopulations of the immune compartment, Notch signaling influences the crosstalk between immune and cancer cells, which may drive drug resistance.

Interestingly, emerging evidence suggests that MDSCs and TAMs, in addition to their canonical role in the immune system, are crucial players in TME-related drug resistance by secreting cytokines, chemokines, and growth factors or by interacting directly with tumor cells^[210]^. Concerning female malignancies, there are only a few recent examples of how Notch signaling is involved in the immune cells-mediated resistance in BC.

It has been shown that the expansion of MDSCs correlates with tumor burden of BC-bearing patients, and the alteration of MDSC-tumor interaction is considered a promising strategy to combat this disease^[242]^. Consistently with this observation, Peng *et al*.^[243] ^demonstrated that MDSCs accelerate tumor progression and increase tumor incidence in NOD-scid IL2Rγ null (NSG) mice *in vivo*. To dissect the underlying molecular mechanism, they observed that MDSCs promote and sustain BCSCs by regulating the crosstalk between STAT3 and Notch signaling in tumor cells. Indeed, MDSCs secretes IL-6, which induces STAT3 phosphorylation, and activates Notch signaling, which enforces IL-6/STAT3 activation, thereby affecting cancer stemness. Interestingly, the inhibition of these two pathways decreased tumor incidence *in vivo*^[243]^.

More recently, Song *et al.*^[244] ^made a step forward linking Notch-mediated MDSC-tumor interaction to drug resistance. By testing the main active component extracted from ginseng (Rg3), the authors documented that the natural compound effectively hampers MDSCs expansion, and this correlates with Notch and STAT3 signaling pathways’ down-modulation, which in turn suppresses cancer stemness and EMT *in vitro* and *in vivo*. Moreover, they showed that Rg3 is able to reverse MDSC-related radio-resistance in BC cell lines co-cultured with MDSCs^[244]^. The above-mentioned studies reported that MDSCs induce Notch signaling in cancer cells to promote drug resistance. Conversely, Liu *et al.*^[245]^ demonstrated that cancer cells shape the TME by inducing the polarization of TAMs towards the anti-inflammatory M2 phenotype. They documented that this polarization is induced by Jagged1 upregulation in ER+ BC cells, and it contributes to the development of resistance to the treatments against ER receptor such as aromatase inhibitors^[245]^.

Collectively, the mechanisms through which Notch signaling and immune cells favor drug resistance must be elucidated, but these recent studies prove that it is an evolving field and may prompt further investigations. Nevertheless, given the different role of Notch signaling in the immune infiltrate, the evaluation of Notch-modulating agents should take into account the subpopulation of cells in the TME.

### Disrupting Notch signaling in tumor vasculature to overcome anti-angiogenic therapy resistance and favor chemotherapy

Female-specific cancers are frequently characterized by exacerbated angiogenesis, which is fundamental for nutrient and oxygen supply to tumor tissue^[246]^. Given that anti-angiogenic therapy has shown promising clinical efficacy, many ongoing clinical trials have been investigating the effects of angiogenesis inhibition in the OC, EC, CC^[247]^, and BC^[248]^ contexts.

In this scenario, VEGF signaling represents the main target, but the efficacy is limited by the non-responsiveness or resistance to the therapy^[249]^. Multiple mechanisms are involved in the afore-mentioned refractoriness^[250]^, including redundancy of the angiogenic signals^[251]^, thereby highlighting the necessity of finding novel strategies to affect the compensatory angiogenic pathways.

Since growing evidence is unraveling the relationship between angiogenesis and Dll4/Notch signaling, Dll4 has emerged as a promising target^[252-254]^. Indeed, over the years, several studies documented that Dll4 inhibition has proved extremely fruitful to overcome anti-VEGF resistance due to the augmented observed effects when combined with anti-VEGF inhibitors^[255-257]^.

Concerning BC, it has been shown that endothelial cells are characterized by high Dll4 expression, and this correlates with adverse prognosis for BC-bearing patients^[258]^. Therefore, these findings pave the way for therapeutic strategies targeting both VEGF and Dll4 pathways. For instance, two independent research groups recently developed dual-specific antibodies targeting both Dll4 and VEGF (ABT-165 and HB-32), obtaining promising results *in vitro* and *in vivo*^[259,260]^.

Similarly, given that high expression of Dll4 in OC correlated with non-responsiveness to anti-VEGF therapy^[261]^, it has been proposed to also block both pathways in this tumor type. Kuhnert *et al.*^[262]^ evaluated the activity of the developed fully human IgG1 monoclonal antibody against Dll4 (REGN421) in OC xenograft models. They observed that REGN421 alone is able to reduce tumor angiogenesis through the disruption of juxtacrine Dll4-Notch1 interactions between endothelial and tumor cells, but the effects are magnified by VEGF blockade^[262,263]^.

Collectively, these data in OC and BC underpin that the simultaneous inhibition of VEGF and Dll4 is a promising therapeutic approach and may warrant further investigations also in the CC and EC contexts.

Notably, an increasing number of studies revealed that angiogenesis is connected to drug resistance^[264]^. Specifically, several studies conducted in female-specific malignancies proved that an effective anti-angiogenic therapy via Notch signaling inhibition favored chemotherapy in OC^[263,265]^ and BC^[259,260]^ preclinical models.

It is worth noting that some interesting results were also obtained in the clinics.

Recently, a recombinant human endostatin (Endostar) was tested on CC-bearing patients in combination with paclitaxel and cisplatin. Since Endostar was developed as a multi-target anti-angiogenic agent, it augmented the cytotoxicity of the chemotherapeutic agents by restoring vascular homeostasis, which was consistent with Dll4 and VEGF inhibition^[266]^. Interestingly, Endostar is currently tested in combination with chemoradiotherapy on CC-bearing patients in two clinical trials in the initial phase (*status*: recruitment) (NCT04121975; NCT03622827).

Moreover, demcizumab (OMP-21M18), an IgG2 humanized monoclonal antibody targeting Dll4, was tested in Phase Ib (NCT01952249) in combination with paclitaxel on platinum-resistant OC, and it produced a positive response^[267]^. Furthermore, a novel bispecific antibody against Dll4/VEGF, navicixumab (OMP-305B83), has completed Phase I (NCT03030287)^[268]^, and it showed antitumor activity in combination with paclitaxel in OC patients, thereby obtaining fast track designation by the FDA.

Overall, this therapeutic strategy needs further development, but it is undoubtedly effective against gynecological cancers.

## CHALLENGES AND FUTURE DIRECTIONS

Many pre-clinical studies have been carried out to elucidate the molecular mechanisms of Notch-mediated drug resistance in female tumors. Although the possibility of manipulating a key regulator of tumoral, stromal, and immune compartments makes this pathway a promising candidate to cope with drug resistance, its inhibition may be challenging.

First, given that Notch receptors are active in healthy tissues, pan-Notch inhibition led to off-target effects in several clinical trials^[269]^. Therefore, research is moving towards Notch-specific targeted therapies to reduce normal tissue toxicity, as clearly described by Majumder *et al.*^[270]^. As a result, more efforts are required in the future to test these novel Notch-modulating agents in synergistic combination with current treatments to effectively tackle Notch-driven resistance.

Second, since Notch signaling is deeply interconnected with other pathways which work in concert to promote drug resistance^[271]^, Notch signaling modulation may be made ineffective by the activation of compensatory pathways. As a result, the dissection of the underlying crosstalks will be necessary to come closer to the above-mentioned goal.

Third, Notch signaling may act differently in cell subpopulations within the same tumor and TME, thereby adding a further layer of complexity. For instance, as mentioned above, Notch signaling plays a pivotal role in shaping tumor immunity, but it can promote a pro- or anti-tumor response, thus questioning the effects of unspecific Notch targeting. These findings underscore the need for selective delivery. In this scenario, nanocarriers may circumvent this obstacle to increase tissue/cell-specific targeting. Interestingly, GSIs and other Notch-modulating agents have begun to be encapsulated in nano-formulations in BC and OC preclinical models^[272]^, thus providing foundations for further studies.

To sum up, taking into account the above-mentioned evidence, further studies should be carried out to achieve higher efficacy and clinical translation of Notch targeting.

## CONCLUSION

Development of resistance severely hampers therapy efficacy and decreases the survival of tumor-bearing patients, including women affected by female-specific cancers. Indeed, given that researchers and clinicians are continuously obliged to face this obstacle, over the past decades, a lot of effort has been put into dissecting this complex phenomenon. Emerging evidence proves the key role played by the evolutionary conserved Notch pathway. In the present review, we describe how Notch signaling contributes to several kinds of resistance, from chemo- and radio-resistance to hormone-based and targeted therapies in female malignancies. The preclinical results obtained in BC, OC, CC, and EC contexts suggest that Notch signaling inhibition can be an effective tool to counteract drug resistance. Nonetheless, given that Notch signaling is broadly involved in tumoral, stromal, and immune compartments, future studies need to focus even more on a way to obtain higher efficacy before translating into the clinics.

## DECLARATIONS

### Copyright

© The Author(s) 2021.
